# Association of Plasma IL-32 Levels and Gene Polymorphisms with Systemic Lupus Erythematosus in Chinese Han Population

**DOI:** 10.1155/2016/2460206

**Published:** 2016-02-29

**Authors:** Yanyun Wang, Bin Zhou, Yi Zhao, Xiuzhang Yu, Yi Liu, Lin Zhang

**Affiliations:** ^1^Laboratory of Molecular and Translational Medicine, Key Laboratory of Birth Defects and Related Diseases of Women and Children of Ministry of Education at Sichuan University, West China Second University Hospital, Sichuan University, Chengdu, Sichuan 610061, China; ^2^Department of Rheumatology and Immunology, West China Hospital, Sichuan University, Chengdu, Sichuan 610041, China; ^3^Department of Obstetrics and Gynecology, West China Second University Hospital, Sichuan University, Chengdu, Sichuan 610061, China

## Abstract

Systemic lupus erythematosus (SLE) is a multisystemic autoimmune disease. IL-32, a secreted protein, has been reported to be associated with several autoimmune diseases. Our preliminary experiment showed different plasma IL-32 levels than that mentioned in a published report on the same population. In order to elucidate the correlation between IL-32 and SLE, we determined the plasma level and two single nucleotide polymorphisms (SNPs) of IL-32 in 152 patients with SLE and 310 healthy controls and analyzed the relationship based on the clinical parameters. The results showed that plasma IL-32 levels in patients with SLE were markedly lower than that in the healthy controls. In the SLE group, patients with detectable IL-32 presented low serum C3 concentrations. Further studies indicated that the rs28372698 SNP was associated with the susceptibility to SLE. Taken together, our results suggested that IL-32 could possibly be a candidate marker to monitor SLE disease stability and screening in future.

## 1. Introduction

Systemic lupus erythematosus (SLE) is a multisystemic autoimmune disease, which involves multiple organ and tissue injuries [1]. The prevalence rate of SLE is about 70 cases per 100,000 people in the Chinese population [2]. Cytokines such as TNF*α*, interleukin 6 (IL-6), IL-27, and IL-12 [3–5] have been reported to be associated with SLE pathogenesis.

IL-32 is a multifunctional secreted protein that plays important roles in antimicrobial pathways [6, 7], cancer [8, 9], and autoimmune diseases, including rheumatoid arthritis (RA), myasthenia gravis, and giant cell arteritis [10–13].

To date, only few studies have focused on the association of SLE and IL-32 [14, 15]. Zhang et al. showed that serum IL-32 level was not statistically different between patients with SLE and healthy controls in the Chinese population [14]. In a preliminary experiment, our team showed that the plasma IL-32 level in healthy controls was markedly different than that observed by Zhang et al. (39.25 (21.00–70.46) pg/mL). Such a difference within the same population seemed abnormal. In the present study, we aimed to better understand the correlation between SLE and IL-32 using serology and immunogenetics in a larger sample size of the Chinese population.

## 2. Materials and Methods

### 2.1. Subjects

One hundred and fifty-two female patients with SLE (16–60 years old) and 310 healthy female controls (18–59 years old) were enrolled in this study. Patients with SLE were recruited from West China Hospital of Sichuan University. The diagnosis of SLE was confirmed according to the 1997-updated SLE criteria wrote by the American College of Rheumatology [16]. SLE disease activity index (SLEDAI) was scored by two independent doctors from the Department of Rheumatology at West China Hospital of Sichuan University. Healthy controls were from a routine health checkup in West China Second University Hospital of Sichuan University. They had no personal or family history of cancer, autoimmune diseases, or other serious diseases. The characteristics of the SLE group were shown in Table 1. This study was approved by the Ethics Committees of West China Hospital of Sichuan University and West China Second University Hospital of Sichuan University. All subjects wrote the informed consent.

### 2.2. Sample Collection

Peripheral blood of all subjects was collected into EDTA-containing vacuum blood collection tube. Samples were centrifuged at 1600 rpm for 10 minutes at 4°C. Plasma was aliquoted and stored at −80°C. Genomic DNA was extracted by a DNA isolation kit from Bioteke (Peking, Beijing, China).

### 2.3. Detection of Plasma IL-32 Level Using an Enzyme-Linked Immunosorbent Assay (ELISA)

Plasma IL-32 levels were measured using a commercially available sandwich ELISA kit (DY3040-05, R&D Systems, Minneapolis, MN, USA) according to the manufacturer's protocol. According to the product manual, the kit recognizes human IL-32*α*, IL-32*β*, and IL-32*γ*. The whole procedure was performed at room temperature (RT). In brief, 96-well microplates were coated with 100 *μ*L per well of the diluted human IL-32 capture antibody. After incubation overnight, plates were washed and then blocked by Reagent Diluent (DY995, R&D Systems, Minneapolis, USA) for 1 hour. The plates were ready for use after washed. Each plasma sample was added to duplicate wells (100 *μ*L per well) and incubated 2 hours. The plates were washed. 100 *μ*L of diluted human IL-32 detection antibody was then added to each well for incubating 2 hours. The plates were washed again. Streptavidin-HRP was added for incubating 20 minutes. Color development was carried out by addition of 100 *μ*L substrate solution (DY999, R&D Systems, Minneapolis, USA). The optical density of each well was determined using a multimode microplate reader (M200, TECAN Infinite, Switzerland) which was set to 450 nm as measurement wavelength and 540 nm as reference wavelength. The four-parameter logistic standard curve was generated using SigmaPlot software version 12.0 (Systat Software Inc., California, USA). The plasma IL-32 concentration was determined by standard curve.

### 2.4. Genotyping of Two Genetic Variants of IL-32

In the present study, two single nucleotide polymorphisms (SNPs), rs12934561 and rs28372698, were genotyped. The experiments were performed as described previously [17]. About 10% of the samples were randomly selected to perform the DNA sequencing analysis. The results were 100% concordant.

### 2.5. Statistical Analyses

The test of normal distribution of plasma IL-32 levels was conducted by SPSS software version 13.0 (SPSS Inc., Chicago, IL, USA). The plasma IL-32 level was described in the form of median (interquartile range). A nonparametric test (Mann-Whiney test) was used to compare plasma IL-32 levels between patients with SLE and healthy controls. Genotype frequencies were determined by directed counting. Hardy-Weinberg equilibrium, genotype, and allele association were performed by chi-square test. Odds ratio (OR) and respective 95% confidence intervals (95% CIs) were used to evaluate the effects of any difference between genotype and alleles. *P* < 0.05 (two-sided test) was considered to be statistically significant.

## 3. Results

### 3.1. Comparison of Plasma IL-32 Levels between Patients with SLE and Healthy Controls

**Figure 1:**
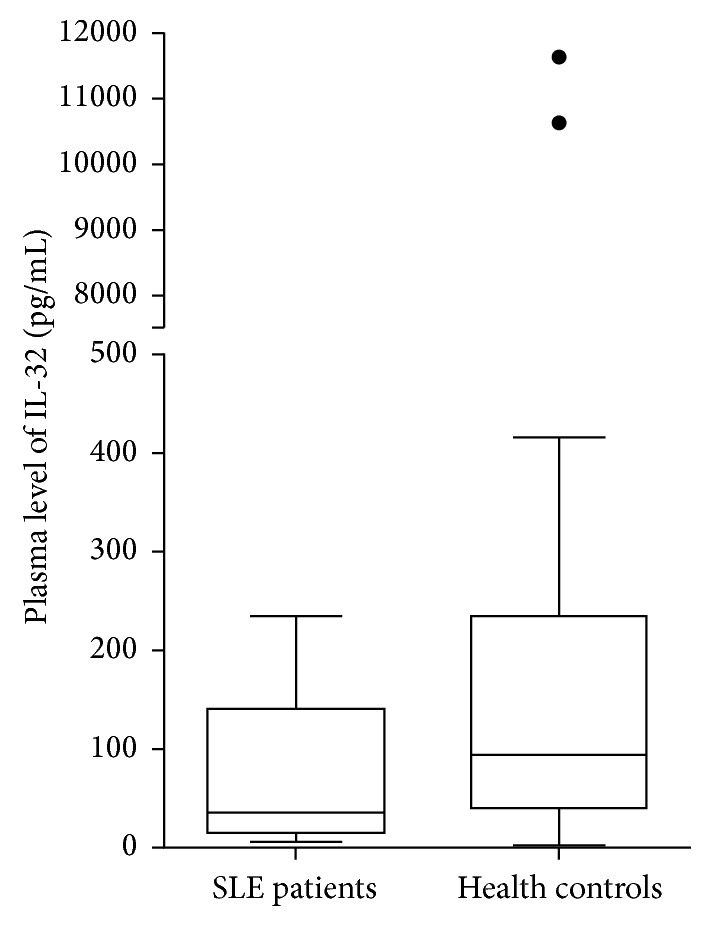
The plasma level of IL-32 in SLE patients and health controls.

Plasma IL-32 levels of 104 patients with SLE and 107 healthy controls were measured by ELISA. Plasma IL-32 levels were detectable in 80 healthy controls and 21 patients with SLE. IL-32 concentration in the plasma was 34.72 (15.45–140.54) pg/mL in patients with SLE and 94.40 (40.21–233.73) pg/mL in the healthy controls (Figure 1). The difference was statistically significant (*P* = 0.02).

Several clinical characteristics were compared between the subgroup of SLE patients with detectable IL-32 and the subgroup in which IL-32 was nondetectable. Serum C3 levels were significantly different between the patients with detectable IL-32 and patients with nondetectable IL-32 (*P* = 0.0215). However, no correlation with SLEDAI score, proteinuria, serum C4, anti-dsDNA, and anti-ANA was observed (Table 2). Among the patients with detectable IL-32, the plasma level of IL-32 was not correlated with SLEDAI score, proteinuria, serum C3, serum C4, anti-dsDNA, and anti-ANA.

Then the relationship between treatment methods and IL-32 level was analyzed. The results explored that IL-32 level was not associated with the treatment methods which the patients were suffering from when the samples were collected (small dose prednisolone plus hydroxychloroquine or prednisolone plus cyclophosphamide or mycophenolate) (data not shown).

### 3.2. Detection of IL-32 SNPs and Susceptibility to SLE

rs12934561 and rs28372698 SNPs were successfully genotyped in all subjects. The genotype frequencies of both SNPs were in agreement with the Hardy-Weinberg equilibrium (*P* < 0.05). The results are presented in Table 3. With regard to rs28372698, the TT genotype was associated with increased risk of SLE (*P* = 0.011, OR = 2.32, and 95% CI = 1.20–4.50 in the recessive model), while the allele frequency was not statistically different between the patients and the healthy controls. Allele and genotype distribution frequencies of rs12934561 showed no significant difference between patients with SLE and healthy controls.

### 3.3. Relationship between Plasma IL-32 Levels and IL-32 SNPs in Patients with SLE

The relationship between plasma IL-32 levels and IL-32 SNPs was analyzed in order to explore if genetic variants could affect plasma IL-32 levels. There was no correlation between the genotype and the plasma IL-32 levels (Table 2).

## 4. Discussion

IL-32 is considered as a proinflammatory cytokine, which is related to IL-1*β*, IL-18, IL-21, and IL-23. It has been reported to play important roles in the pathogenesis of various autoimmune diseases. Gui et al. showed that the level of IL-32 in the plasma was significantly associated with RA disease activity [10]. Another study suggested that IL-32 might be correlated with the pathogenesis or immunoregulation of myasthenia gravis [11].

Only two studies on the association between SLE and IL-32 were found in the PubMed database (http://www.ncbi.nlm.nih.gov/pubmed/). Zhang et al. showed that serum IL-32 concentrations in patients with SLE were not different from that in the healthy controls in a Chinese population [14]. Although both our study and that of Zhang et al. focused on the Chinese population, our study was markedly different from that of Zhang et al. In the present study, plasma IL-32 levels in patients with SLE were lower than those of the controls. There are six IL-32 isoforms (IL-32*α*, IL-32*β*, IL-32*γ*, IL-32*δ*, IL-32I, and IL-32*ζ*) [18]. The detected IL-32 isoforms were not described clearly in the study by Zhang et al. Thus, we cannot be sure that the difference observed in the same population was caused by different indices of detection.

We speculate that the lower plasma IL-32 level in the SLE group may be associated with drug treatment. In fact, the concentration of serum IL-17 could be reduced after cyclophosphamide treatment for 4 weeks in patients with SLE and IL-17 is known to affect the expression of IL-32 [19, 20]. Majority of the patients with SLE (enrolled in our study) were taking medicine for at least six months. Therefore, IL-32 levels may have changed. Additionally, IL-32 was shown to be associated with the chemotherapy-related bone marrow cytotoxicity [21]. The arrest of bone marrow was the most common immunosuppressant side-effect for patients with SLE. This might be another possible reason for decreased IL-32 levels. Although the treatment methods did not associate with the IL-32 detection and level in our present study, in the future, the SLE patients without treatment would be needed to be enrolled in the follow-up study. It could be helpful in better exploring the correlation between treatment and IL-32.

In the present study, plasma IL-32 was detectable in only 20.2% of the patients. Inoue et al. detected the serum level of IL-32*γ* in 51 patients with SLE and 15 healthy controls. The results demonstrated that IL-32 was detectable in only 3 patients and led to the speculation that IL-32*γ* possibly contributes to the pathogenesis of renal diseases in patients with SLE [15]. In our study, no correlation between plasma IL-32 level and urinary proteins or complicated lupus nephritis was observed. However, serum C3 levels tended to be low in patients with detectable IL-32, suggesting that IL-32 could possibly be a candidate marker to monitor SLE disease stability and screening in future. As the more sensitive detection methods were improved, the nondetectable samples in the present study might be detected; the difference and association among SLE group might be explored more clearly.

Genetic factors, such as SNPs, have been reported to be associated with the corresponding serum level [22–24]. In order to explore if the different plasma IL-32 levels in patients with SLE were correlated with the genetic effect, two SNPs (rs12934561 and rs28372698) were detected in this study. rs12934561 is a C/T single nucleotide variation in an intron, which is associated with acute lung injury and endometrial cancer [17, 25]. rs28372698, a promoter SNP, is associated with the risk of gastric cancer and endometrial cancer [17, 26]. We analyzed the correlation between the genotype of the patients for these two SNPs and plasma IL-32 levels. The result showed no significant association between plasma IL-32 levels and the genotype.

Interestingly, the TT genotype of rs28372698 was found to be associated with increased risk of SLE in the recessive model. However, the role of this polymorphism with regard to the function of IL-32 remains unknown due to the lack of corresponding reports. Future studies are warranted to investigate this question.

In summary, the present study explored the correlation between IL-32 polymorphism and SLE for the first time and provided new data regarding IL-32 levels in the plasma of a Chinese population. Taken together, our results suggested that IL-32 could possibly be a candidate marker to monitor SLE disease stability and screening in future.

## Figures and Tables

**Table 1:** Characteristics of the patients.

	Patients
Mean duration of disease (months)	1–156
SLEDAI (*n*)	
0–9 score	94
*⩾*10 score	58
Lupus nephritis (*n*)	
Yes	105
No	47
24 h urinary protein (*n*)	
<0.5 g	94
*⩾*0.5 g	58
C3 (*n*)	
<0.785 g/L	96
*⩾*0.785 g/L	56
C4 (*n*)	
<0.145 g/L	69
*⩾*0.145 g/L	83
Anti-dsDNA (*n*)	
Positive	65
Negative	87
Anti-ANA (*n*)	
Positive	144
Negative	8

**Table 2:** Clinical Characteristics and genotype of two SNPs of patients whose plasma levels of IL-32 were measured.

	IL-32 detectable (*n* = 21)	IL-32 not detectable (*n* = 83)	*P* value
Age (mean ± SD), years	37 ± 10.58	35.17 ± 11.12	
Mean duration of disease (months)	6–144	1–156	
SLEDAI (*n*)			
0–9 score	16	62	1.000
*⩾*10 score	5	21	
Lupus nephritis (*n*)			
Yes	16	59	0.7880
No	5	24	
24 h urinary protein (*n*)			
<0.5 g	13	59	0.4360
*⩾*0.5 g	8	24	
C3 (*n*)			
<0.785 g/L	20	58	**0.0215** ^*∗*^
*⩾*0.785 g/L	1	25	
C4 (*n*)			
<0.145 g/L	15	51	0.4560
*⩾*0.145 g/L	6	32	
rs28372698 genotype (*n*)			
AA	7	39	0.3045
AT	12	32	
TT	2	12	
rs12934561 genotype (*n*)			
TT	13	30	0.1010
TC	5	33	
CC	3	20	

^*∗*^Statistically significant.

**Table 3:** Distribution of IL-32 SNPs in SLE patients and healthy controls and their association with SLE susceptibility.

Model	rs28372698					rs12934561				
	Genotype	Controls	Patients	OR (95% CI)	*P* value	Genotype	Controls	Patients	OR (95% CI)	*P* value
		*N* = 310	*N* = 152				*N* = 310	*N* = 152		
Codominant	AA	150 (0.484)	69 (0.454)	1.00 (reference)	**0.038** ^*∗*^	TT	103 (0.332)	63 (0.414)	1.00 (reference)	0.189
	AT	141 (0.455)	63 (0.414)	0.97 (0.64–1.47)		TC	149 (0.481)	61 (0.401)	0.67 (0.43–1.03)	
	TT	19 (0.061)	20 (0.132)	2.29 (1.15–4.56)		CC	58 (0.187)	28 (0.184)	0.79 (0.46–1.37)	
Dominant	AA	150 (0.484)	69 (0.454)	1.00 (reference)	0.545	TT	103 (0.322)	63 (0.414)	1.00 (reference)	0.084
	AT/TT	160 (0.516)	83 (0.546)	1.13 (0.76–1.67)		TC/CC	207 (0.668)	89 (0.586)	0.70 (0.47–1.05)	
Recessive	AA/AT	291 (0.939)	132 (0.868)	1.00 (reference)	**0.011** ^*∗*^	TT/TC	252 (0.813)	124 (0.816)	1.00 (reference)	1.000
	TT	19 (0.061)	20 (0.132)	2.32 (1.20–4.50)		CC	58 (0.187)	28 (0.184)	0.98 (0.60–1.62)	
Overdominant	AA/TT	169 (0.545)	89 (0.586)	1.00 (reference)	0.411	TT/CC	161 (0.519)	91 (0.6)	1.00 (reference)	0.108
	AT	141 (0.455)	63 (0.414)	0.85 (0.57–1.26)		TC	149 (0.481)	61 (0.4)	0.72 (0.49–1.07)	
	*Allele*									
	A	441 (0.711)	201 (0.661)	1.26 (0.94–1.70)	0.129	T	355 (0.573)	187 (0.615)	0.84 (0.63–1.11)	0.217
	T	179 (0.289)	103 (0.339)			C	265 (0.427)	117 (0.385)		

*Notes*. *N*, the number of individuals. ^*∗*^Statistically significant.
